# Phase Transformation Principle and Magnetite Grain Growth Law in the Magnetization Sintering Process of Oolitic Hematite Ore

**DOI:** 10.3390/ma18153649

**Published:** 2025-08-03

**Authors:** Hanquan Zhang, Xunrui Liu, Lei Xie, Tiejun Chen, Fan Yang, Bona Deng

**Affiliations:** 1 School of Resources & Safety Engineering, Wuhan Institute of Technology, Wuhan 430074, China; 13477022296@139.com (H.Z.); lxrfaiz@126.com (X.L.); ywlwtg@163.com (L.X.); huahe735@gmail.com (F.Y.); 2 School of Resources & Environmental Engineering, Wuhan University of Science and Technology, Wuhan 430081, China; ctj_56@163.com

**Keywords:** oolitic hematite ore, magnetization sintering, magnetite, reduction, grain growth

## Abstract

Oolitic hematite ore represents a significant iron resource, but its utilization is challenging due to the complex multi-layered circular structure of hematite ore, which makes it difficult to be reduced. This study systematically investigated the phase transformation principle and magnetite grain growth law during the magnetization sintering of oolitic hematite ore, aiming to establish optimal conditions for efficient hematite ore to magnetite conversion. The results demonstrated that both elevated temperature and prolonged reduction duration significantly enhanced the reduction efficiency of hematite (Fe_2_O_3_) to magnetite. The optimal sintering conditions were determined to be 700 °C for 45 min, under which the magnetite content and Fe/O atomic ratio in the roasted products peaked at approximately 68% and 0.8%, respectively. However, temperatures exceeding 800 °C proved detrimental to magnetite formation, as further reduction to Fe_X_O phases occurred. Notably, appropriate temperature elevation promoted substantial magnetite grain growth. When the sintering temperature increased from 600 °C to 700 °C, both the absolute and relative thickness of the magnetite layer exhibited remarkable enhancement, expanding from 9.52 μm to 76.76 μm and from 5.99% to 50.33%, respectively. Furthermore, comparative analysis revealed that a high sintering temperature for a short time was more effective for magnetite particle growth than a low temperature for a long time in the magnetization process of oolitic hematite ore.

## 1. Introduction

In recent decades, the global steel industry has undergone rapid development, and the demand for iron ores is huge. With a depletion of high-quality iron ores, the utilization of refractory iron ores is imperative to support the development of the iron industry [1,2,3]. Oolitic hematite ore is a typical refractory iron ore with a huge reserve and distributed worldwide [4,5,6]. It was found that the deposit of high-phosphorus oolitic hematite ore is approximately 4 billion tons [7,8,9]. Upgrading and utilizing this type of ore can effectively provide a resource supply for the steel industry.

It is difficult to beneficiate the oolitic hematite ore mainly due to the fine-grained dissemination of hematite (Fe_2_O_3_) and the complex mineral structure. The diameter of hematite (Fe_2_O_3_) contained is mainly 5–10 μm, which means that the hematite (Fe_2_O_3_) cannot be effectively recovered. Oolitic hematite ore is primarily composed of oolitic, gravel, granular, laminated, and block structures, of which the oolitic structure is the most typical. The oolitic structure is formed owing to the multi-layer concentric circular bands composed of hematite ore, oolitic green clay, calcite, and collophane [10,11,12,13,14].

Many beneficiation techniques have been applied to treat the oolitic hematite ore. Gravity separation and flotation processes were intensively used to beneficiate oolitic hematite ore. However, the gravity separation process is only applicable to the iron-rich oolites due to the difficulty in mineral dissociation. The flotation process has the disadvantage of a complicated reagent process, high reagent costs, and dilemmas in handling concentrates and tailings [15,16,17,18]. As a result, the pyrometallurgical process was further developed for the optimization of oolitic hematite ore.

Direct reduction–magnetic separation is an efficient method to recover iron from the oolitic hematite ore, since the hematite (Fe_2_O_3_) has been effectively reduced to metallic iron, which can be collected by a magnetic separation process and further directly used in the iron-making industry [19]. Nevertheless, the process faces challenges such as high energy consumption and difficulties in removing phosphorus. Thus, the magnetization sintering–magnetic separation technique is proposed. It is not necessary for the hematite (Fe_2_O_3_) to be reduced to metallic iron, but it must be transferred to magnetite, which can also be recovered by a magnetic separation process. The energy consumption of magnetization sintering is much lower than the direct reduction process, and prospected to be the most effective method for the beneficiation of the oolitic hematite ore.

The growth of magnetite grains during the magnetization sintering process of hematite ore is a temperature-driven diffusion mechanism. Under high-temperature conditions, the diffusion of iron and oxygen ions leads to the nucleation and outward expansion of magnetite grains, usually forming a continuous magnetite shell around the unreduced core. This diffusion-controlled grain growth process significantly affects the grain size, morphology, and magnetic characteristics of the final product, and these parameters are critical to the industrial magnetic separation process [20,21,22,23].

The key to magnetization sintering lies in the effective transformation of hematite (Fe_2_O_3_) into magnetite. However, this process is difficult control, since hematite (Fe_2_O_3_) can be easily reduced to other minerals if the reduction conditions are not suitable. If the temperature is higher than about 560 °C, FeO can be produced as one of the products. The reduction process of hematite (Fe_2_O_3_) mainly undergoes the following procedures: Fe_2_O_3_ → Fe_3_O_4_ → FeO → Fe. It can be seen that the magnetite is an intermediate state in the reduction process of hematite (Fe_2_O_3_). Both under-reduction and over-reduction are unfavorable to the formation of magnetite [24,25,26,27]. Therefore, it is essential to investigate the growth principle of magnetite in the magnetization sintering process of oolitic hematite ore for highly efficient iron recovery.

Researchers have conducted many studies on the growth principles of iron particles in the direct reduction process [28,29,30,31]. Li et al. [32] measured and statistically analyzed the size of metallic iron particles through a scanning electronic microscope, and found out that probably prolonging the reduction time and increasing the reduction temperature was beneficial to the growth of metallic iron particles. Zhang et al. [33] confirmed that the adjacent newly formed magnetite was aggregated to form larger magnetite grains during the magnetization sintering of oolitic hematite ore, but the growth mechanism was not explicit. 

In this study, the phase transformation principle of iron minerals and the growth rules of newly formed magnetite particles during the coal-based magnetization sintering process of cryptocrystalline oolitic hematite ore was investigated. The optimized sintering conditions for the formation of magnetite was ascertained to provide a support for the further efficient utilization of the oolitic hematite ore.

## 2. Materials and Methods

### 2.1. Materials

**Figure 1:**
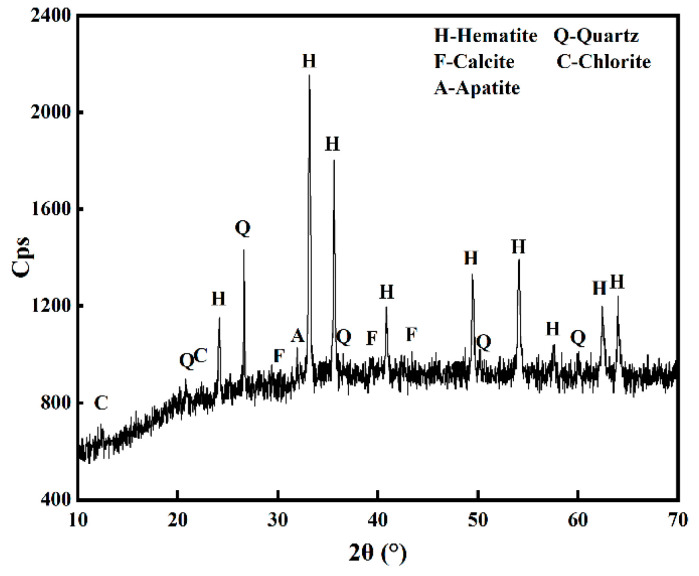
XRD pattern of oolitic hematite ore.

In this work, the cryptocrystalline oolitic hematite ore samples were collected from Enshi, Hubei province. Chemical phase analysis was carried out to determine the distribution of iron in the samples. Table 1 shows the iron distribution in the dominant mineral phases in the oolitic hematite ore. It was confirmed that the iron is mainly enriched in hematite (Fe_2_O_3_) and limonite. The iron content was 48.3 wt.%, accounting for 96.79% of the total iron in the oolitic hematite ore. The X-ray diffraction patterns of the oolitic hematite ore are shown in Figure 1. It was indicated that the dominant mineral in the oolitic hematite ore was hematite (Fe_2_O_3_), followed by small proportions of quartz, calcite, apatite, and chlorite.

In the experiment, coal powder with a particle size of −2 mm was selected as the reducing agent, and its industrial analysis results are shown in Table 2. The fixed carbon content of the coal is 52.59 wt.%, indicating that it has good reducing properties and can provide a stable and continuous reducing atmosphere during the magnetization sintering process. The volatile content is 29.07 wt.%, and the moderate and high volatile content is conducive to the rapid release of combustible gas at the initial stage of heating and improves the reducing property of the reaction system. The ash content is 10.11 wt.%, which is low and helps to reduce slag formation and interference with the reduction reaction of iron minerals, thereby improving the reduction efficiency. The sulfur content is only 0.64 wt.%, and the low sulfur content helps to inhibit the sulfidation reaction of iron minerals and avoid sulfur contamination of the quality of the finished iron concentrate. In summary, the coal has good pyrolysis characteristics and reducing ability, and is suitable for the magnetization sintering experiment of this study.

### 2.2. Magnetization Sintering

The ore sample was mixed with coal powder and placed in a covered crucible, which was then roasted in a muffle furnace at a preset temperature. In order to ensure a good reducing atmosphere during the sintering process, bituminous coal with a fixed carbon content of 52.59% and a volatile content of 29.07% was selected as a reducing agent (see Table 2). The higher fixed carbon content can continuously release CO and CO_2_ during the heating process to form a reducing atmosphere, while the higher volatile content further helps to generate reducing gases at high temperatures. After the sintering, the sample was immediately sealed and placed in wet coal powder to cool to prevent re-oxidation. The roasted ore after cooling was further ground for analysis and testing of the mineral composition.

### 2.3. Test Techniques

The mineral composition of the oolitic hematite ore was determined by an X-ray diffraction (XRD) analyzer (Rigaku SmartLab SE, Tokyo, Japan) the proportion of different minerals contained in the sample can further be calculated through the Rietveld analysis function. The LEICA DMI electron microscope was utilized to capture microscopic images of the main iron minerals. The light gray represented hematite (Fe_2_O_3_) while the dark gray denoted magnetite. Based on the color difference, the particle size of magnetite could be distinguished. The growth trend of newly formed magnetite particles under different sintering conditions could be deduced by statistical principles.

## 3. Results

### 3.1. Phase Transformation of Oolitic Hematite Ore During Magnetization Sintering Process

The main factors influencing the magnetization sintering process of the oolitic hematite ore were the sintering temperature and sintering time. In order to understand the transformation principle of the iron minerals during the magnetization sintering process of oolitic hematite ore, XRD and the semi-quantitative analysis of mineral compositions were conducted on the roasted products under different thermal conditions. All samples were roasted in a reducing atmosphere.

**Figure 2:**
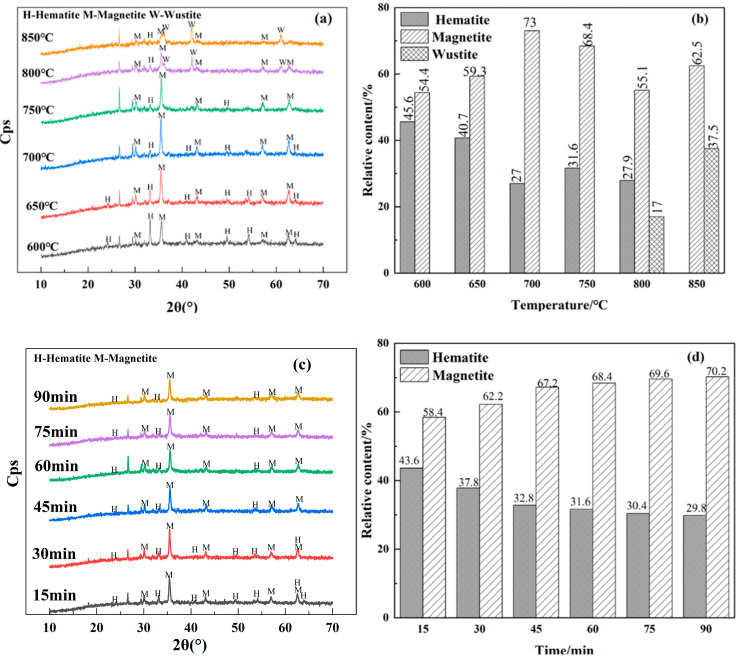
XRD patterns and the contents of the contained mineral phases of the roasted oolitic hematite ore: (**a**) XRD patterns roasted at different temperatures and roasting time of 60 min; (**b**) corresponding contents of mineral phases roasted at different temperatures; (**c**) XRD patterns roasted at different times and roasting temperature of 700 °C; (**d**) contents of mineral phases roasted at different times.

Figure 2 illustrates the mineral compositions and the relative content of hematite (Fe_2_O_3_) and magnetite of the roasted products at various sintering conditions. The effect of the sintering temperature on the phase transformation process was studied, and the results are shown in Figure 2a,b. The sintering time was fixed at 60 min. It was shown that, with an increase in the sintering temperature, the iron minerals were gradually reduced as the diffraction intensity and peak area of hematite (Fe_2_O_3_) was reduced, while the peak diffraction intensity and the content of magnetite were increased. Magnetite was formed at approximately 600 °C, and the content of magnetite reached maximum as the sintering temperature was 700 °C. As the sintering temperature was further increased, the peak intensity of magnetite and the magnetite content started decreasing, owing to the generation of FexO. At the sintering temperature of 850 °C, hematite (Fe_2_O_3_) was fully reduced and its content could not be detected. The iron minerals in the reduced product were transformed into magnetite and FexO. It was confirmed previously that the existence of FexO would cause the poor low-intensity magnetic separation efficiency of iron. Thus, 700 °C was the optimum sintering temperature for the recovery of magnetite.

At a sintering temperature of 700 °C, sintering with coal, the effect of the sintering time on the formation of magnetite was investigated, and the results are shown in Figure 2c,d. The peak intensity of the hematite (Fe_2_O_3_) phase decreased with increasing sintering time, while that of magnetite gradually increased. At a sintering time of 15 min, magnetite was already generated, and its content was increased with a prolonged sintering time. The content of magnetite was increased evidently as the sintering time was increased from 15 min to 45 min, and slightly increased as the sintering time was prolonged from 45 min to 90 min. It was ascertained that 45 min was sufficient for the formation of magnetite in the magnetization process of oolitic hematite ore.

### 3.2. Grain Growth Law of Generated Magnetite

#### 3.2.1. Statistical Analysis of Grain Thickness of Magnetite

Since the shape of oolitic particles in the images were irregular, the thickness of the magnetite layer was difficult to measure. In each cross-sectional image, a straight line was drawn perpendicular to the magnetite layer interface, and the length of its intersection with the magnetite layer was measured as the grain thickness. The discreteness of the data was evaluated by calculating the standard deviation of the measurement results. The results showed that the standard deviation was small, indicating that the method had good repeatability. In this study, the line segment method was introduced to measure the magnetite thickness layer.

**Figure 3:**
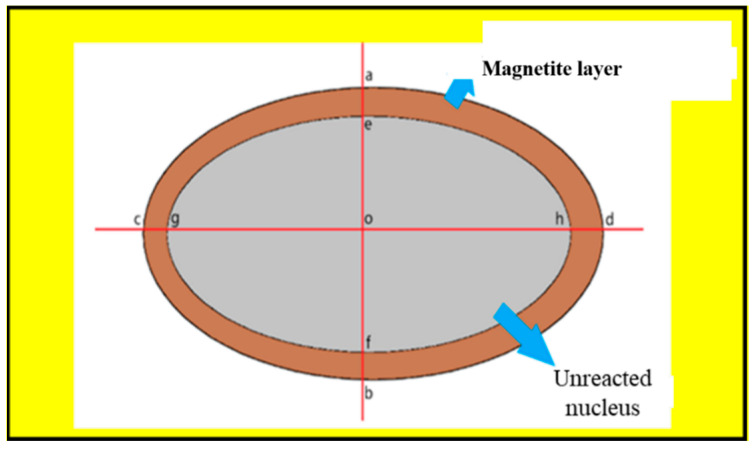
Schematic diagram of roasted ore particles.

Figure 3 shows the schematic diagram of oolitic hematite ore after magnetization sintering, which can be divided into a magnetite layer and an unreacted core. The number of magnetite particles selected for each condition was N ≥ 50. The thickness of magnetite layer can be calculated by the following:

Thickness of magnetite layer of single magnetite particle:

d = (ae + bf + cg + dh)/4(1)

Total thickness of magnetite layer under various conditions:

(2) $$D=\sum_{i=1}^{n}d_{i}$$

Relative thickness of magnetite layer for a single magnetite particle:

(3) $$r_{i}=\frac{\frac{ae+bf}{ab}+\frac{cg+dh}{cd}}{2}$$

Total relative thickness of magnetite layer under various conditions:

(4) $$R=\sum_{i=1}^{n}r_{i}$$

#### 3.2.2. Effect of Sintering Temperature

**Figure 4:**
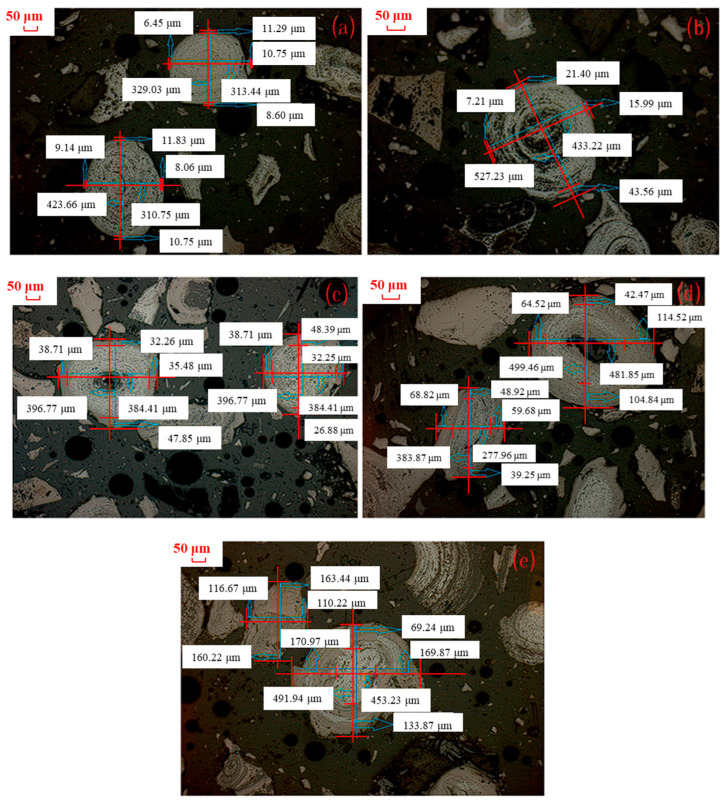
Microstructure of products sintered at different temperatures and sintering time of 60 min: (**a**) 600 °C; (**b**) 650 °C; (**c**) 700 °C; (**d**) 750 °C; (**e**) 800 °C microstructure.

As the formation principle of magnetite has already been investigated in the above Section 3.1, the effect of sintering temperature and sintering time on the growth law of magnetite particles was studied in this section. Figure 4 presents microscopic images of the roasted products obtained from the magnetization process of the cryptocrystalline oolitic hematite ore at various temperatures with a sintering time of 60 min, sintering with coal. Due to the non-uniformity of the magnetite particle size, the thickness of the magnetite layer of single magnetite particles was not representative. The relative thickness of magnetite layer of different magnetite particles was thus calculated. Table 3 shows the statistical and calculation results regarding the thickness and relative thickness of the magnetite layer in the micrograph of the roasted product (with a statistical sample size of ≥50 magnetite particles).

**Figure 5:**
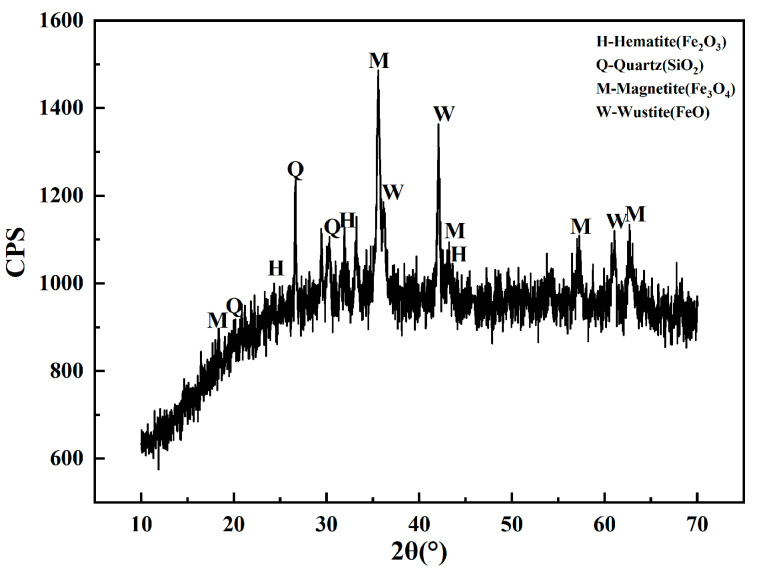
XRD of coal-based sintering products at 800 °C.

As observed in Table 3, the thickness of a generated magnetite particle increased from 9.52 μm to 76.76 μm with an increase in the sintering temperature from 600 °C to 750 °C, while the relative thickness was raised from 5.99% to 50.33%. Upon further increase in the sintering temperature to 800 °C, the thickness of magnetite particles decreased to 68.91 μm, while the relative thickness slightly increased to 52.87%. This was primarily because the generated Fe_3_O_4_ was further reduced to FeO at a higher sintering temperature, which was confirmed by the XRD patterns of the roasted products at 800 °C, as shown in Figure 5.

It was evident that FexO was exactly generated at the sintering temperature of 800 °C, as its diffraction patterns were detected. In addition, impurity components in the ore, such as phosphorus and silicon dioxide, showed significant stability during the roasting process. XRD analysis showed that phosphorus mainly existed in the form of apatite, and its diffraction peak was still clearly visible after roasting at 800 °C, indicating that this component did not undergo significant decomposition or transformation during the roasting process, showing high thermal stability. Silicon dioxide mainly existed in the form of quartz, which did not undergo significant phase change or chemical reaction before and after roasting, and still existed in a stable inert phase.

**Figure 6:**
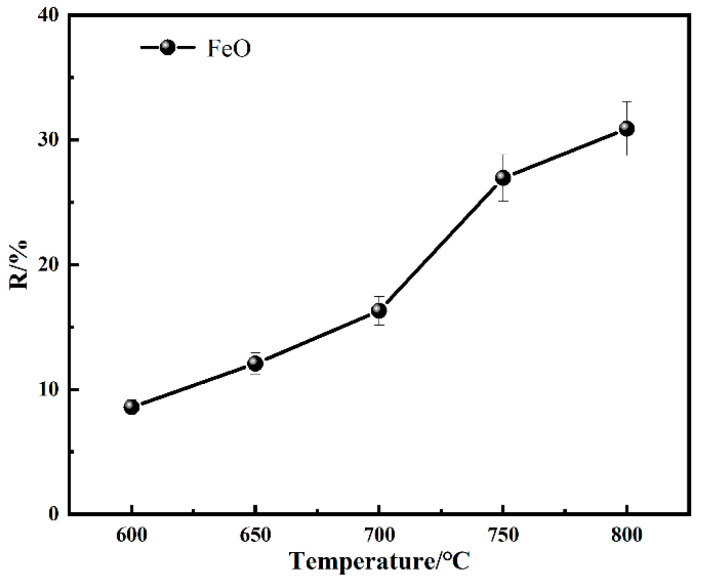
Changes in FeO content at different coal-based sintering temperatures.

Figure 6 illustrates the variation in the FeO content in the roasted products at various sintering temperatures. At the sintering temperature of 600–700 °C, sintering with coal, only a small amount of Fe_2_O_3_ was transformed into Fe_3_O_4_; thus, the FeO content was relatively low. As the sintering temperature was increased to 750 °C, the Fe_3_O_4_ was formed rapidly and, as a result, the FeO content became higher. With the sintering temperature further increased to 800 °C, the generated magnetite was further reduced to FexO, which resulted in a deceleration of the generation rate of FeO.

**Figure 7:**
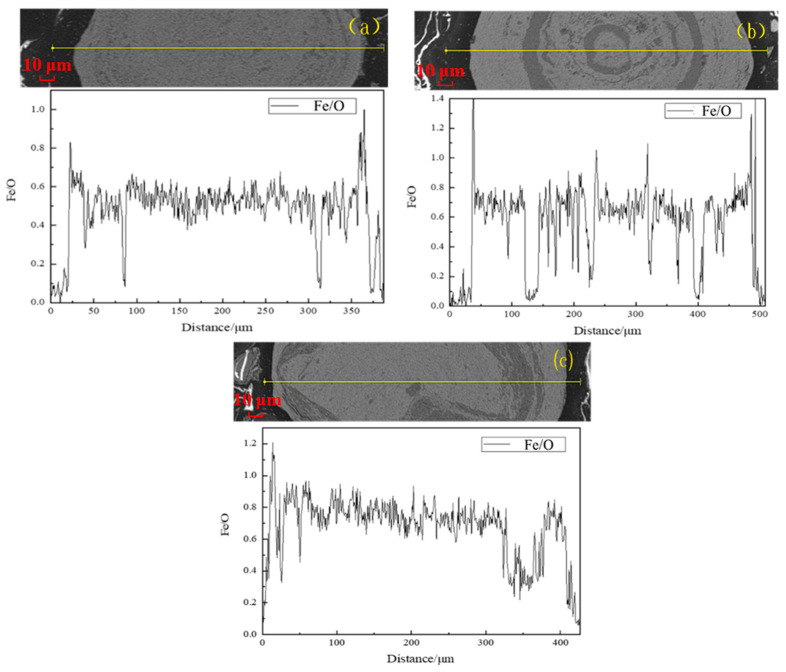
The variation in Fe/O in products sintered at different temperatures and time of 60 min: (**a**) 600 °C; (**b**) 700 °C; (**c**) 800 °C.

Besides the content of FeO, the Fe/O atomic ratio in the reduced products also can be used to characterize the grain growth of the newly generated magnetite particles. At the sintering temperature range for the generation of magnetite, a high Fe/O atomic ratio represented a high Fe^2+^ content, which further demonstrated a good reduction of hematite ore to magnetite. Although the Fe/O ratio can be a simplified indicator of the degree of reduction of magnetite, this method still has certain limitations. Iron in natural ores may exist in different mineral phases, such as iron silicate, apatite inclusions, etc., and its reduction path is different from that of pure iron oxide, which may affect the accuracy of the Fe/O ratio. Therefore, the use of the Fe/O ratio needs to be combined with mineral phase analysis and other physical property testing methods to obtain a more comprehensive and reliable evaluation result. SEM-EDS analysis with line scanning was conducted on the roasted products to analyze the variation in the Fe/O atomic ratio at different sintering conditions. The results are exhibited in Figure 7.

As depicted in Figure 7, the Fe/O atomic ratio was gradually increased with the increase in the sintering temperature. The Fe/O atomic ratio was raised from approximately 0.6% at 600 °C to the maximum of about 0.8% at 700 °C. As the sintering temperature increased further to 800 °C, the Fe/O atomic ratio varied little, which indicated that an obvious over-reduction of magnetite occurred. It was confirmed that the magnetization reduction principle of hematite (Fe_2_O_3_) was positive with the growth law of the generated magnetite layer under various sintering temperatures.

#### 3.2.3. Effect of Sintering Time

**Figure 8:**
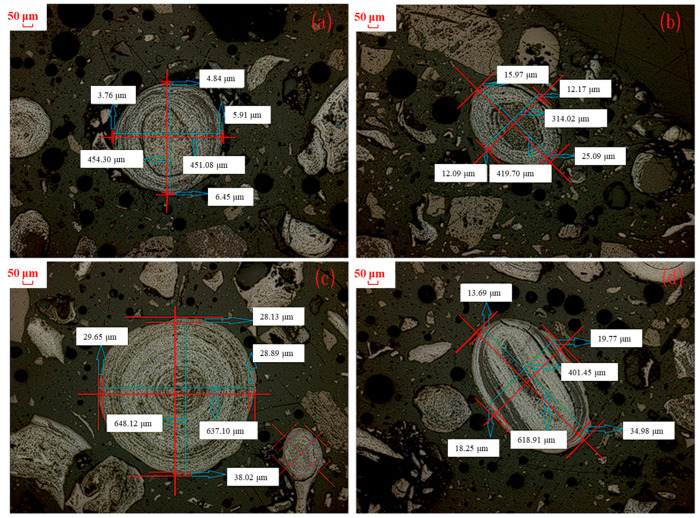
Microstructure diagram of sintered products at different sintering times and sintering temperature of 600 °C: (**a**) 60 min; (**b**) 90 min; (**c**) 120 min; (**d**) 150 min.

Besides the sintering temperature, sintering time is also crucial to the conversion of the cryptocrystalline oolitic hematite ore in the magnetization sintering process. The effect of the sintering time on the grain growth law of the newly generated magnetite was studied, and the results at a low temperature of 600 °C and a high temperature of 800 °C were analyzed, respectively. Figure 8 shows the microscopic images of calcined cryptocrystalline oolitic hematite ore treated with a relatively longer sintering time from 60 min to 150 min at a sintering temperature of 600 °C. The thickness of the magnetite layer and its relative thickness were calculated, and the results are shown in Table 4.

It was indicated that the oolitic hematite ore was not completely reduced, as the inner core remained unreduced. With the sintering time increased from 60 min to 120 min at 600 °C, the thickness of the magnetite layer gradually increased from 11.28 μm to 31.35 μm. As the sintering time was prolonged to 150 min, the thickness of the magnetite layer was decreased. At the same time, it was seen that the relative thickness of the generated magnetite layer increased from 6.53% to 19.05% as the sintering time was increased from 60 min to 120 min, and decreased to 17.35% at the sintering time of 150 min. The results indicated that the grain growth rate at 600 °C was relatively slow, and a longer sintering time was not favorable for the reduction of hematite (Fe_2_O_3_), since the coal was not sufficient to maintain the reduction atmosphere in this situation. The generated magnetite was oxidized again, as the sintering time was too long, and the thickness of the magnetite layer was thus decreased.

**Figure 9:**
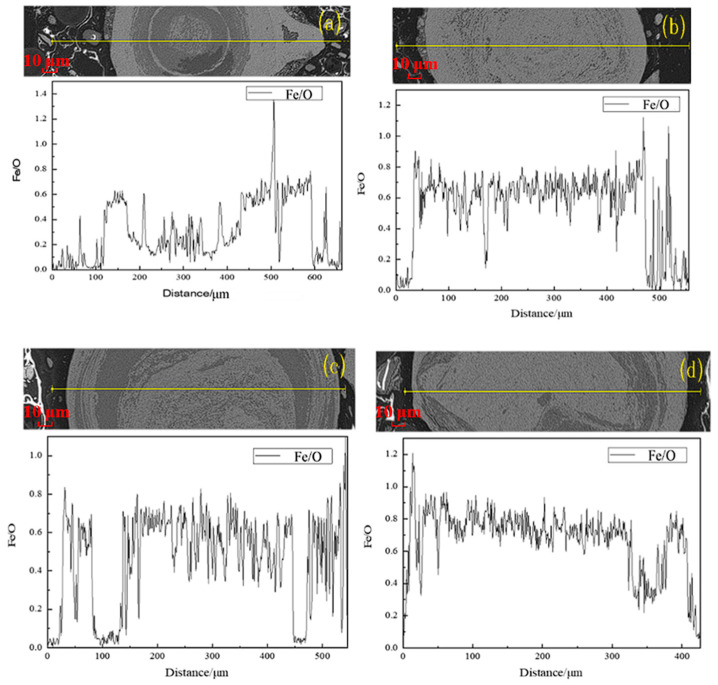
Changes in Fe/O atomic ratios in products with different sintering times at sintering temperature of 600 °C: (**a**) 60 min; (**b**) 90 min; (**c**) 120 min; (**d**) 150 min.

To further investigate the effect of sintering time on the reduction of hematite (Fe_2_O_3_) at a temperature of 600 °C, SEM-EDS analysis was performed on the roasted product, and Fe/O atomic ratios at different sintering times are presented in Figure 9. The value of Fe/O was about 0.6% with a sintering time of 60 min at a sintering temperature of 600 °C, and increased to 0.8% as the sintering time was prolonged to 120 min. But, when the sintering time was further increased to 150 min, the Fe/O atomic ratio started to decrease, and the value was 0.7%. The results were aligned with the thickness variation in the generated magnetite layer at 600 °C.

**Figure 10:**
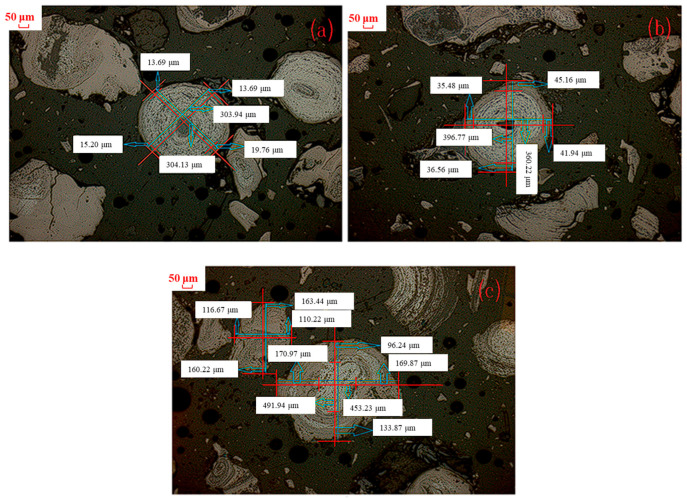
Microstructure of coal-based sintered products at different sintering times with a sintering temperature of 800 °C: (**a**) 20 min; (**b**) 40 min; (**c**) 60 min.

Figure 10 displays the microscopic images of cryptocrystalline oolitic hematite ore after being reduced at different sintering times at a sintering temperature of 800 °C. The thicknesses of the generated magnetite layer were calculated and are listed in Table 5. As the sintering time increased from 20 min to 40 min, the thickness of the generated magnetite layer was gradually increased from 33.84 μm to 48.43 μm. Subsequently, the thickness exhibited an accelerated growth rate, and the value increased to 68.91 μm as the sintering time was 60 min. At the same time, the relative thickness of the generated magnetite increased from 20.43% to 52.87% as the sintering time increased from 20 min to 60 min, which was positive with the variation in the magnetite thickness layer. Both the thickness and relative thickness of the magnetite layer confirmed successful hematite (Fe_2_O_3_) reduction without magnetite over-reduction at 800 °C, as long as the sintering time was properly regulated.

**Figure 11:**
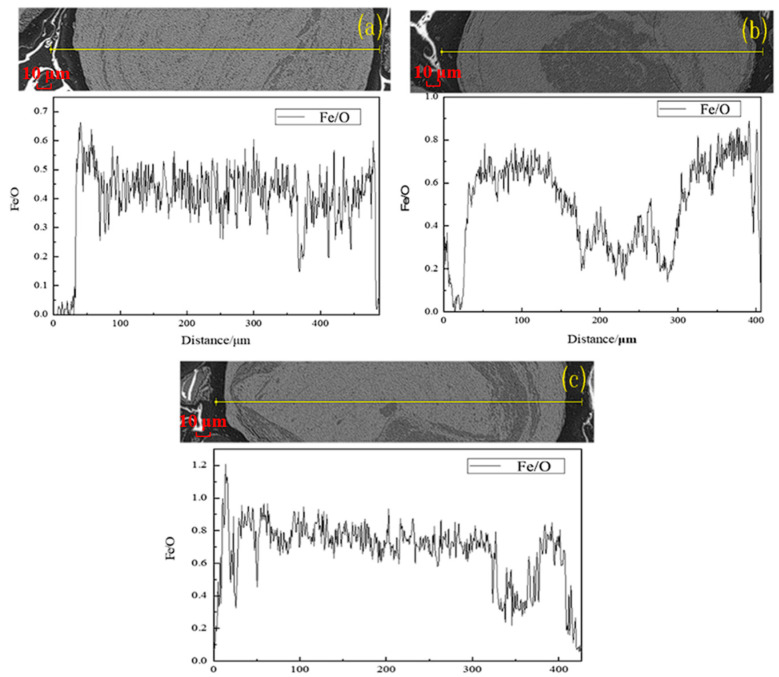
Changes in Fe/O atomic ratios in products with different sintering times at sintering temperature of 800 °C: (**a**) 20 min; (**b**) 40 min; (**c**) 60 min.

The SEM-EDS analysis of the roasted products at a sintering temperature of 800 °C and different sintering times is presented in Figure 11. The ratio of Fe/O increased from 0.5% to approximately 0.8% as the sintering time was increased from 20 min to 60 min at 800 °C, suggesting a good reduction of hematite ore.

## 4. Conclusions

(1) Magnetite was generated when the sintering temperature was higher than 600 °C in the magnetization process of the oolitic hematite ore. The sintering temperature of 700–750 °C was desirable for the generation of magnetite, since the relative content of magnetite in the roasted product reached the maximum. Higher temperatures above 800 °C were not favorable for the formation of magnetite, as it was further reduced to FexO.
(2) Relatively long sintering times were beneficial to the reduction of hematite (Fe_2_O_3_). After optimization, the sintering time of 45 min proved sufficient for magnetite generation in the magnetization process of oolitic hematite ore when the sintering temperature was 700 °C.
(3) The grain growth rate of the generated magnetite gradually accelerated as the sintering temperature was increased from 600 °C to 750 °C, and as the thickness and relative thickness of the generated magnetite layer was increased from 9.52 μm to 76.76 μm and 5.99% to 50.33%, respectively. At the same time, the Fe/O atomic ratio was increased from approximately 0.6% at 600 °C to the maximum of about 0.8% at 700 °C. A further increase in the sintering temperature would hardly enhance the magnetite thickness and Fe/O atomic ratio in the roasted product.
(4) High-temperature sintering for a short time proved to be more favorable than low-temperature sintering for a long time for the growth of generated magnetite particles in the magnetization process of oolitic hematite ore. The relative thickness and growth rate of the magnetite layer at a sintering temperature of 800 °C and sintering times of 20–60 min proved superior to those obtained at a sintering temperature of 600 °C and sintering times of 60–150 min.

## Figures and Tables

**Table 1:** Distribution of iron in dominant iron mineral phases in the oolitic hematite ore /wt.%.

Iron Mineral Phases	Fe in Magnetite	Fe in Siderite	Fe in Sulfide	Fe in Hematite Ore and Limonite	Fe in Ferric Silicate	Total
Content /%	0.07	0.56	0.28	48.30	0.69	49.90

**Table 2:** Mass fraction of the main components in the coal /wt.%.

Moisture Content	Volatile	Ash Content	Fixed Carbon	Sulfur Content
7.7	29.07	10.11	52.59	0.64

**Table 3:** Calculation results of magnetite layer thickness generated at different coal-based sintering temperatures.

Time/min	Temperature/°C	Thickness Layer of Magnetite/μm	Relative Thickness Layer of Magnetite/%
60	600	9.52 ± 0.48	5.99 ± 0.32
	650	25.90 ± 1.30	17.06 ± 0.85
	700	64.50 ± 2.60	35.64 ± 1.78
	750	76.76 ± 3.10	50.33 ± 2.20
	800	68.91 ± 2.75	52.87 ± 2.10

**Table 4:** Thickness and relative thickness of magnetite layer generated during coal-based sintering at 600 °C and different sintering times.

Temperature/°C	Time/min	Thickness Layer of Magnetite/μm	Relative Thickness Layer of Magnetite/%
600	60	11.28 ± 0.58	6.53 ± 0.36
	90	18.91 ± 0.76	11.17 ± 0.55
	120	31.35 ± 1.10	19.05 ± 0.85
	150	29.43 ± 1.00	17.35 ± 0.80

**Table 5:** Thickness and relative thickness of the magnetite layer formed at 800 °C during coal-based sintering and at different sintering times.

Temperature/°C	Time/min	Thickness Layer of Magnetite/μm	Relative Thickness Layer of Magnetite/%
800	20	33.84 ± 1.20	20.45 ± 0.85
	40	48.43 ± 1.45	38.58 ± 1.10
	60	68.91 ± 1.80	52.87 ± 1.32

## Data Availability

The original contributions presented in this study are included in the article. Further inquiries can be directed to the corresponding authors.
